# Evaluation of the physiological activity of venom from the Eurasian water shrew *Neomys fodiens*

**DOI:** 10.1186/s12983-017-0230-0

**Published:** 2017-09-30

**Authors:** Krzysztof Kowalski, Paweł Marciniak, Grzegorz Rosiński, Leszek Rychlik

**Affiliations:** 1 Department of Systematic Zoology, Institute of Environmental Biology, Adam Mickiewicz University, Umultowska 89, 61-614 Poznań, Poland; 2 Department of Animal Physiology and Development, Institute of Experimental Biology, Faculty of Biology, Adam Mickiewicz University, Umultowska 89, 61-614 Poznań, Poland

**Keywords:** Mammalian venom, Natural toxins, *Neomys fodiens*, Toxicity in vitro, Salivary glands, Shrews, *Sorex araneus*

## Abstract

**Background:**

Animal toxins can have medical and therapeutic applications. Principally, toxins produced by insects, arachnids, snakes and frogs have been characterized. Venomous mammals are rare, and their venoms have not been comprehensively investigated. Among shrews, only the venom of *Blarina brevicauda* has been analysed so far, and blarina toxin has been proven to be its main toxic component. It is assumed that *Neomys fodiens* employs its venom to hunt larger prey. However, the toxic profile, properties and mode of action of its venom are largely unknown. Therefore, we analysed the cardio-, myo- and neurotropic properties of *N. fodiens* venom and saliva of non-venomous *Sorex araneus* (control tests) in vitro in physiological bioassays carried out on two model organisms: beetles and frogs. For the first time, we fractionated *N. fodiens* venom and *S. araneus* saliva by performing chromatographic separation. Next, the properties of selected compounds were analysed in cardiotropic bioassays in the *Tenebrio molitor* heart.

**Results:**

The venom of *N. fodiens* caused a high decrease in the conduction velocity of the frog sciatic nerve, as well as a significant decrease in the force of frog calf muscle contraction. We also recorded a significant decrease in the frog heart contractile activity. Most of the selected compounds from *N. fodiens* venom displayed a positive chronotropic effect on the beetle heart. However, one fraction caused a strong decrease in the *T. molitor* heart contractile activity coupled with a reversible cardiac arrest. We did not observe any responses of the insect heart and frog organs to the saliva of *S. araneus*. Preliminary mass spectrometry analysis revealed that calmodulin-like protein, thymosin β-10, hyaluronidase, lysozyme C and phospholipase A2 are present in the venom of *N. fodiens*, whereas thymosin β4, lysozyme C and β-defensin are present in *S. araneus* saliva.

**Conclusion:**

Our results showed that *N. fodiens* venom has stronger paralytic properties and lower cardioinhibitory activity. Therefore, it is highly probable that *N. fodiens* might use its venom as a prey immobilizing agent. We also confirmed that *S. araneus* is not a venomous mammal because its saliva did not exhibit any toxic effects.

**Electronic supplementary material:**

The online version of this article (10.1186/s12983-017-0230-0) contains supplementary material, which is available to authorized users.

## Background

Many animal toxins have been discovered in the last century [1]. Even several species of toxic birds, such as *Pitohui kirhocephalus* or *Ifrita kowaldi*, have been discovered [2]. However, despite the availability of new proteomic, genomic and chromatographic separation techniques, most venomous and poisonous animals remain unstudied [3–5]. Principally, toxins produced by insects, arachnids, snakes and frogs have been investigated thus far [3, 6]. Venoms produced by mammals remain mostly uncharacterized [3, 5, 6].

Only a few mammalian species, such as the short-tailed shrew, two species of water shrews and two species of solenodons (Soricomorpha), as well as the platypus (Monotremata), are proven to produce venoms [7–9]. Additionally, according to recent research, three species of vampire bats (Chiroptera) and three species of lorises (Primates) are considered venomous as well [5, 9–11]. Only the platypus venom has been comprehensively studied with a focus on its composition, function and evolution [12]. Some studies on toxic components and function of the vampire bat and loris (especially the slow loris) venom have been performed [10, 11]. Likewise, the biological activities of the venoms produced by few insectivorous mammals (from the order Soricomorpha) have been reported [7–9]. In general, studies on the structure of venomous glands and the toxic activity of solenodon venom are scarce [13, 14]. Among shrews, only the venom from the short-tailed shrew *Blarina brevicauda* has been analysed thus far [8, 15]. The main toxic component of this venom is blarina toxin (BLTX) — a serine protease with tissue kallikrein-like activity [8]. Moreover, blarinasin was purified and characterized in *B. brevicauda* venom [15]. However, despite the chemical similarity to BLTX, this protease is completely devoid of toxic activity. It is particularly surprising that the knowledge on venoms from two species of water shrews, *Neomys fodiens* and *N. anomalus*, quite common in Europe, is extremely poor [7, 9]. The toxic properties of *N. fodiens* and *N. anomalus* venoms from the submandibular salivary glands have been reported a few times in the past [16, 17]. Therefore, it is highly probable that, similar to *B. brevicauda*, the venoms of these two species contain compounds with toxic activity.

In the present paper, we aimed to analyse the profile and toxic activity of the venom from the Eurasian water shrew *N. fodiens*. The paralytic (neuro- and myotoxic) and cardiotoxic properties of the water shrew venom were identified by performing physiological bioassays on two model experimental animals: the mealworm beetle *Tenebrio molitor* and frogs (*Rana temporaria* and *Pelophylax* sp.). Additionally, control tests with the saliva of a non-venomous species, the common shrew *Sorex araneus*, were carried out.

## Results

### In vitro effects of the water shrew venom and its fractions on the heart contractile activity

**Fig. 1:**
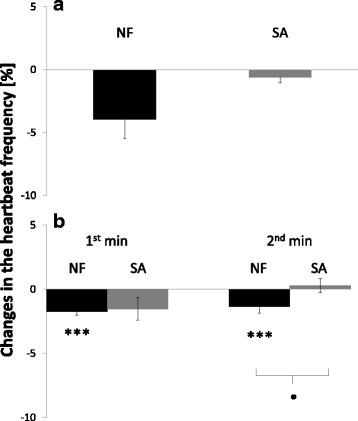
Decrease in the heartbeat frequency after the application of *N. fodiens* venom (NF) and *S. araneus* saliva (SA). **a** Treatment of the *T. molitor* heart with 10 μl of *N. fodiens* venom extract and 10 μl of *S. araneus* saliva. **b** Treatment of the frog heart with 40 μl of venom or saliva (registration of the heart contractile activity for 2 min). Statistically significant differences are indicated by asterisks (NF: Wilcoxon-test in **a**, *n* = 32; paired Student’s *t*-test in **b**, *n* = 30; SA: paired Student’s *t*-test in **a**, *n* = 40; Wilcoxon-test in **b**, *n* = 30) for intraspecific comparisons (*** *p* < 0.001) and black dots (unpaired Student’s *t*-test) for comparisons between NF and SA (• *p* < 0.05)

**Fig. 2:**
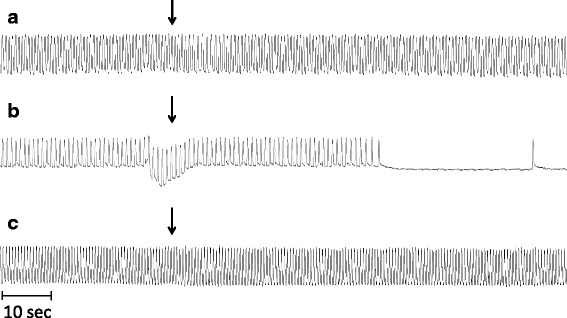
Cardiomyograms displaying changes in the *T. molitor* heartbeat frequency after treatment with *N. fodiens* venom and *S. araneus* saliva. **a** Application of 10 μl of venom extract of *N. fodiens*. **b** Application of fraction no. 5 from *N. fodiens* venom. **c** Treatment with 10 μl of saliva extract of *S. araneus*. The sample application is indicated by an arrow

**Fig. 3:**
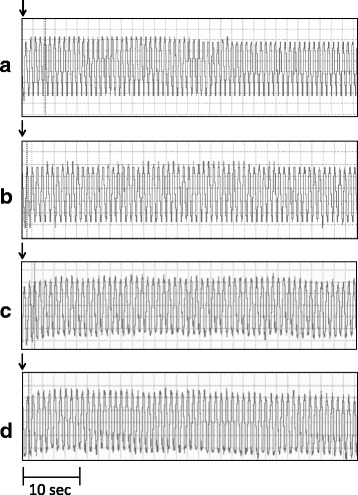
Cardiomyograms displaying changes in the frog heartbeat frequency after treatment with *N. fodiens* venom and *S. araneus* saliva. The heart activity after application of 40 μl of Ringer’s solution (control) (**a**, **c**), *N. fodiens* venom (in the 1^st^ minute) (**b**), and *S. araneus* saliva (in the 1^st^ minute) (**d**). The sample application is indicated by an arrow

The application of venom extract of the water shrew on the semi-isolated insect heart caused a small decrease in the heart contractile activity (−3.95% ± 1.51; Figs. 1a and 2a). After the application of *N. fodiens* extract on the frog heart, a small but significant decrease in the heart contractile activity was observed. During the 1^st^ minute after venom application, the frequency of the heartbeat decreased by 1.73% ± 0.30 (Figs. 1b and 3b), whereas during 2^nd^ minute by 1.36% ± 0.50 (Fig. 1b). There was no change in the insect heart activity after the application of saliva extract from the common shrew (−0.62% ± 0.40; Figs. 1a and 2c). Additionally, after the application of the common shrew saliva on frog heart we did not record any changes in the heart contractile activity (1^st^ minute: −1.53% ± 0.87; 2^nd^ minute: 0.28% ± 0.55; Figs. 1b and 3d). Moreover, the venom of the water shrew displayed a stronger negative chronotropic effect on the frog heart than *S. araneus* saliva. However, this result was significant only in the 2^nd^ minute after sample application (Fig. 1b).

**Fig. 4:**
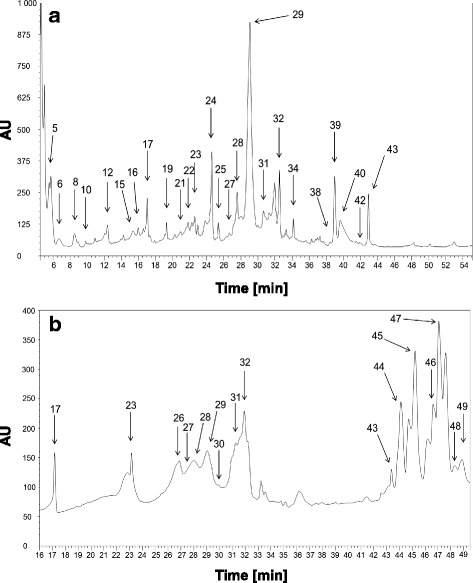
Chromatograms displaying separation of the methanolic extract of the saliva of *N. fodiens* (**a**) and *S. araneus* (**b**). The analysed fractions are indicated by arrows

From 55 fractions obtained after the RP-HPLC (reverse phase high-performance liquid chromatography) separation of *N. fodiens* venom, 25 fractions were selected to analyse the cardioactivity in bioassays with the *T. molitor* heart (Fig. 4a). In the case of *S. araneus*, 16 fractions were assayed (Fig. 4b).

**Fig. 5:**
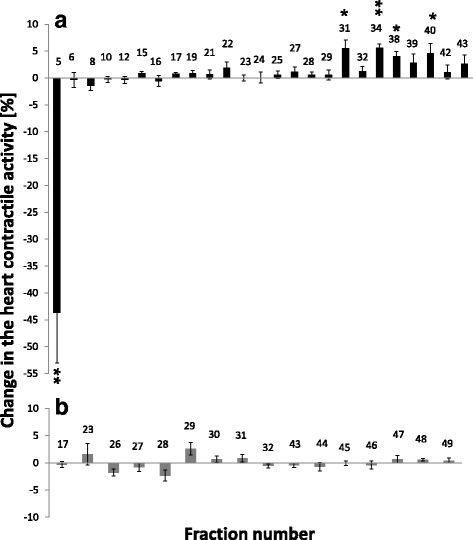
Effects of fractions from *N. fodiens* venom (**a**) and *S. araneus* saliva (**b**) on the *T. molitor* heartbeat frequency. Statistically significant changes in the heart contractile activity are indicated by asterisks (Wilcoxon-test after Bonferroni correction: * *p’* < 0.05, ** *p’* < 0.01)

Among compounds from *N. fodiens* venom, fraction no. 5 caused a strong and highly significant decrease in the insect heart contractile activity (−43.7% ± 9.40; Fig. 5a). Moreover, in most cases, we observed a short and reversible cardiac arrest after the application of fraction no. 5 on the insect heart (Fig. 2b). Most of the other components (especially fractions no. 31, 34, 38 and 40) caused a small but significant increase in the heartbeat activity (Fig. 5a).

Among the components from the saliva of *S. araneus*, no fractions displayed cardioactivity (Fig. 5b). We did not observe any changes in the heartbeat frequency of the adult *T. molitor*. Only the application of fraction no. 29 caused an almost significant change. However, an increase in the insect heart contractile activity after the application of this fraction was very low (2.58% ± 1.14; Fig. 5b). The results of statistical analysis of this part are available in the Additional files section (see Additional file 1).

### In vitro effects of venom from the water shrew on the frog calf muscle activity

**Fig. 6:**
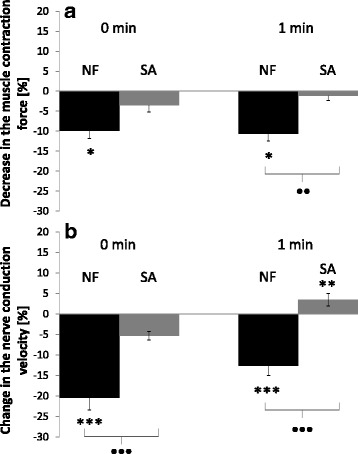
Changes in the contraction force of the frog calf muscle (**a**) and sciatic nerve conduction velocity (**b**). In both tests, 40 μl of extract from *N. fodiens* venom (NF) and 40 μl of *S. araneus* saliva (SA) were applied. Statistically significant differences are indicated by asterisks (NF: paired Student’s *t*-test in **a**, n = 30; Wilcoxon-test in **b**, *n* = 32; SA: Wilcoxon-test in **a**, *n* = 30; paired Student’s *t*-test in **b**, *n* = 30) for intraspecific comparisons (* *p* < 0.05, *** *p* < 0.001) and black dots (**a**: Mann-Whitney *U*-test; **b**: unpaired Student’s *t*-test in 0 min and Mann-Whitney *U*-test in 1 min after) for comparisons between NF and SA (• *p* < 0.05, ••• *p* < 0.001)

The venom of the water shrew displayed a significant influence on the contractions of frog calf muscle. Immediately after the application of the venom on the muscle, the force of the muscle contraction was reduced by 9.86% ± 1.98. One minute after venom application, the muscle activity was still significantly decreased (−10.66% ±1.78; Fig. 6a). We did not observe such an influence of the saliva of the common shrew on the frog calf muscle contraction activity (Fig. 6a). Immediately after saliva application, the force of the muscle contraction was reduced by 3.50% ± 1.66. One minute later, it was reduced by 1.10% ± 1.19. Furthermore, *N. fodiens* venom displayed a stronger negative influence on muscle activity than saliva of the common shrew. This difference was almost significant immediately after sample application, whereas it was highly significant 1 min later (Fig. 6a).

### In vitro effects of venom from the water shrew on the frog sciatic nerve activity

The venom of *N. fodiens* displayed a very strong negative effect on the frog sciatic nerve activity. Immediately after venom application, we observed a highly significant decrease in the nerve conduction velocity (−20.45% ± 2.98; Fig. 6b). Similarly, 1 min after venom application, the activity of the frog nerve was still significantly decreased (−12.67% ± 2.28; Fig. 6b). We did not record such a change immediately after the application of *S. araneus* saliva on the frog sciatic nerve (−5.33% ± 1.08; Fig. 6b). By contrast, 1 min after treatment with the saliva of the common shrew, there was a small but significant increase in the nerve conduction velocity (3.43% ± 1.48; Fig. 6b). Moreover, the venom of the water shrew displayed a stronger negative neurotropic effect on the frog sciatic nerve than the saliva of the common shrew. This difference was highly significant both immediately after venom application and 1 min later (Fig. 6b).

### Proteomic pre-identification of proteins from *N. fodiens* venom and *S. araneus* saliva

Preliminary mass spectrometry (MS) analysis enabled us to predict some protein/peptide molecules that are present in *N. fodiens* venom and might be involved in its toxic activity (Table 1). Calmodulin-like protein, thymosin β-10, hyaluronidase and β-nerve growth factor were found in the methanolic extract from *N. fodiens* venom. Among the compounds determined in fractions, obtained by chromatographic separation, were hyaluronidase (fraction no. 39), cystatin C (fraction no. 5), coagulation factor VIII (fractions no. 5 and 39), lactyloglutathione lyase (fraction no. 39) and inhibitor of leech-derived tryptase (fraction no. 34). Additionally, lysozyme C was found in fraction no. 5, whereas phospholipase A2 and calmodulin-like protein were present in fraction no. 40. Phospholipase A2 (protein sequence coverage: 4%) was also detected in the methanolic extract of *N. fodiens*.

**Table 1:** Pre-identification of proteins from *N. fodiens* venom and *S. araneus* saliva based on tandem mass spectrometry analysis

Sample	Protein name	Species	Accession	Matched peptides	Protein sequence coverage [%]	Ion score	m/z	Identified peptides	Possible toxic activity
*Neomys fodiens*									
extract	calmodulin-like protein	*Mus musculus*	Q9D6P8	25	37	64	955	K.EAFSLFDK.D	Acute and chronic effects on cardiac function by regulation of the intracellular Ca^2+^ concentration [38]
						37	4086	R.SLGQNPTEAELQGMVNEIDKDGNGTVDFPEFLTMMSR.K + Oxidation (M)	
						57	4102	R.SLGQNPTEAELQGMVNEIDKDGNGTVDFPEFLTMMSR.K + 2 Oxidation (M)	
						97	1351	K.MKDTDSEEEIR.E	
						81	1367	K.MKDTDSEEEIR.E + Oxidation (M)	
						65	1092	K.DTDSEEEIR.E	
	hyaluronidase-2	*Bos taurus*	Q8SQG8	14	8	24	980	HKMPLDPK	Facilitates spread of other venom proteins [36, 42]
	thymosin β-10	*Bos taurus*	P21752	11	11	22	862	KTETQEK	Improves cardiac function, promotes vascularization and contractility in heart tissue [52, 53]
	β-nerve growth factor	*Mus musculus*	P01139	8	6	68	1153	K.LQHSLDTALR.R	Unknown [36, 54]
						37	764	R.RLHSPR.V	
fraction no. 5	cystatin-C	*Rattus norvegicus*	P14841	14	8	28	1207	GTHTLTKSSCK	Inhibits cysteine proteases, failures in biological mechanisms controlling protease activities may led to many diseases such as neuro-degeneration or cardiovascular diseases [39]
	coagulation factor VIII	*Sus scrofa*	P12263	18	5	22	1427	ISALGKSAAGPLASGK	Acts as an anti-hemophilic factor [55]
	lysozyme C-1	*Sus scrofa*	P12067	6	8	24	930	YWCNDGK	Involved in an antimicrobial defence [10, 56]
fraction no. 31	hyaluronidase PH-20	*Myotis brandtii*	gi|521028001	14	11	47	1152	KDIEFYIPK	See above
fraction no. 34	chain E, leech-derived tryptase inhibitor Trypsin Complex	*Sus scrofa*	gi|3318722	48	21	177	2210	R.LGEHNIDVLEGNEQFINAAK.I	Prolongs the blood clotting time by thrombin and trypsin inhibition [57]
						115	2282	K.IITHPNFNGNTLDNDIMLIK.L	
						93	1045	K.LSSPATLNSR.V	
						77	841	R.VATVSLPR.S	
						108	1515	K.SSGSSYPSLLQCLK.A	
						98	1051	K.APVLSDSSCK.S	
fraction no. 39	coagulation factor VIII	*Sus scrofa*	P12263	19	7	23	1427	ISALGKSAAGPLASGK	See above
	lactyloglutathione lyase	*Rattus norvegicus*	Q6P7Q4	17	25	78	1264	K.DFLLQQTMLR.I	Involved in inflammation [58]
						54	1028	K.KSLDFYTR.V	
						44	900	K.SLDFYTR.V	
						57	976	K.RFEELGVK.F	
						65	2288	K.GLAFVQDPDGYWIEILNPNK.M	
fraction no. 40	phospholipase A2	*Oryctolagus cuniculus*	P14422	6	6	27	1002	FAKFLSYK	Exhibits cardio-, myo- and neurotoxicity, as well as pro- and anticoagulant effects [40, 41]
	calmodulin-like protein	*Mus musculus*	Q9D6P8	14	5	25	1367	MKDTDSEEEIR	See above
*Sorex araneus*									
extract	thymosin β-10	*Rattus norvegicus*	P63312	7	34	56	862	K.KTETQEK.N	See above
						37	734	K.TETQEK.N	
						48	875	K.ETIEQEK.R	
						77	1031	K.ETIEQEKR.S	
	coagulation factor XI	*Mus musculus*	Q91Y47	10	11	21	846	MICAGYK	Involved in blood clotting [59]
fraction no. 23	thymosin β-4	*Oryctolagus cuniculus*	P34032	69	88	46	1245	M.ADKPDMAEIEK.F	See thymosin β-10
						47	1652	M.ADKPDMAEIEKFDK.S + Oxidation (M)	
						34	862	K.KTETQEK.N	
						38	734	K.TETQEK.N	
						83	1371	K.TETQEKNPLPSK.E	
						89	1512	K.NPLPSKETIEQEK.Q	
						43	875	K.ETIEQEK.Q	
						72	1348	K.ETIEQEKQAGES.-	
	cystatin-C	*Rattus norvegicus*	P14841	14	8	27	1207	GTHTLTKSSCK	See above
fraction no. 28	cystatin-C	*Rattus norvegicus*	P14841	11	6	43	1207	K.GTHTLTKSSCK.N	See above
	lysozyme C-1	*Sus scrofa*	P12067	10	19	22	787	AWVAWR	See above
	kallikrein 1-related peptidase b24	*Mus musculus*	Q61754	10	6	33	1204	K.DKSNDLMLLR.L	Might act as an inflammatory agent (increasing vascular permeability and lowering blood pressure) [8, 9]
fraction no.29	cystatin-C	*Rattus norvegicus*	P14841	14	8	27	1207	GTHTLTKSSCK	See above
	α-amylase 1	*Mus musculus*	P00687	8	11	25	780	DYVRTK	Unknown
	β-defensin 7	*Mus musculus*	Q91V70	8	11	24	760	FQIPEK	Exhibits a significant myo- and neurotoxic activity, modifying voltage-sensitive Na^+^ channels, resulting in a potent analgesic effect [36]

Thymosin β-10 and coagulation factor XI were present in the saliva of *S. araneus*, whereas cystatin-C was found in fractions no. 23, 28 and 29 (Table 1). Additionally, we found thymosin β-4 in fraction no. 23, lysozyme C in fraction no. 28 and β-defensin in fraction 29, as well as kallikrein 1-related peptidase in fraction no. 28. Nonetheless, these results should be treated with caution as further analyses (including obtaining a larger quantity of tissue material or more precise saliva purification) are required.

## Discussion

At the end of the 50s and 60s of the last century, Pucek [16, 17] discovered that *N. fodiens* and *N. anomalus* produce in their salivary glands a potent venom that is toxic to mammals such as mice, voles and rabbits. The strongest toxic effects of saliva were revealed after intracerebral (mice and voles) and intravenous injections (rabbits). Following injection, experimental animals were overcome by a general depression and showed symptoms that included excessive urination, irregular respiration, paralysis of the hind limbs, convulsions and, finally, death [16, 17]. Similar symptoms were observed when the saliva of *B. brevicauda* was administered to mice, voles, rabbits and cats [7, 18, 19]. As noted by Lawrence [20], the venom of *B. brevicauda* displays predominantly neurotoxic activity. Our results also prove that the venom from the water shrew has stronger paralytic (neuro- and myotoxic) activity, whereas its cardiotropic effect on the frog and beetle heartbeat frequency is relatively small. At first, it might seem surprising, but it becomes more comprehensible when the food and metabolic requirements of shrews are taken into consideration.

It is well known that shrews must consume a large amount of food to meet their particularly high metabolic demands [21, 22]. Although shrews are mainly insectivorous, it has been repeatedly reported that they can also consume vertebrates, even larger than themselves, such as murid rodents, fish, frogs or lizards [23–26]. Therefore, it might be expected that venom could be a useful tool in catching and paralysing larger prey [7, 8]. Hunting larger prey might provide the food supply for a longer time, especially if the prey remains paralysed and decomposes considerably slowly. According to the optimal foraging theory, the hoarding of a food supply can save energy and time spending on prey foraging and catching. Furthermore, it might also minimize the risk of predation [27–29]. The food-storing habits of shrews have also been reported [21, 30–33]. According to Rychlik and Jancewicz [33], the water shrew usually hoards larger prey than other shrew species. Therefore, it is highly probable that *N. fodiens* can employ its venom to seize and immobilize such prey. Our results also support the hypothesis that *N. fodiens* venom may be a useful tool in the handling and paralysing of larger prey.

However, it should be noticed that Pucek [16, 17] observed the conspicuous effects of the water shrew venom after intracerebral and intravenous injections. We do not deny these results, but it seems that the recorded symptoms could partly result from mechanical damage caused by the insertion of a needle into the brain rather than from venom injection. Therefore, in our study, we analysed the responses of specific organs to venom application in in vitro bioassays instead of an examination of the whole organism response. Moreover, Pucek [16, 17] and Pearson [18] characterized only the properties of crude extracts from the salivary glands of shrews. In the present work, besides the investigation of the activity of salivary extract from the venom, for the first time, we examined the toxic activity of selected compounds from *N. fodiens* venom and *S. araneus* saliva fractionated by chromatographic separation.

In general, our results indicate that many components from *N. fodiens* venom have a weak but positive chronotropic influence on *T. molitor* heart contractile activity. However, fraction no. 5 displayed a strong negative chronotropic effect on the heartbeat frequency coupled with a short and reversible cardiac arrest. It has been reported that *B. brevicauda* can use the venom as an insect-immobilizing agent [20, 34]. Thus, due to the cardioinhibitory activity of at least one molecule contained in the *N. fodiens* venom, we suppose that the water shrew can also employ its venom to hunt certain invertebrate prey, such as larger beetles, diplopods or crickets.

Most animal venoms comprise a mixture of bioactive compounds, such as proteins and peptides, salts or amino acids [35–37]. Therefore, it is highly probable that certain neurotoxins or proteins, similar to BLTX, may be present in the venom of *N. fodiens*. We are aware that obtaining a larger quantity of tissue material (by pooling extracts from salivary glands collected from at least 25 specimens) or more precise purification of fractionated samples are required to identify specific toxic molecules from the water shrew venom. Nevertheless, preliminary MS analysis did enable us to predict specific toxic compounds that presumably are present in *N. fodiens* venom and might be involved in its toxic activity. We suggest that calmodulin-like protein or cystatin C might contribute to the decrease in the frequency of the heartbeat. Zhang et al. [38] found that calmodulin may have acute and chronic effects on cardiac function. Cystatin-C has been proven to inhibit cysteine proteases and lead to neuro-degeneration and cardiovascular diseases [39]. In addition, we found lysozyme C and phospholipase A2 (PLA2) in fractions no. 5 and 40 from *N. fodiens* venom. Dufton [7] also reported lysozyme from the saliva of the water shrew, whereas PLA2 is widely distributed among elapid and viperid snake venoms [40, 41]. It has been proven that PLA2 molecules exhibit various pharmacological effects, such as cardio-, myo- and neurotoxicity, as well as pro- and anticoagulant effects [40, 41]. Therefore, it is possible that phospholipase A2 might also be responsible for the paralytic and cardiotropic symptoms recorded by us, but these conclusions are, by far, more speculative. Hyaluronidase, which is commonly present in animal venoms [42], might promote the spreading of these and the other components from *N. fodiens* venom [36, 42]. Additionally, it is highly possible to reveal kallikrein-like proteins (similar to BLTX) in the venom of the water shrew, especially because, in the present work, we found kallikrein 1-related peptidase in the saliva of the common shrew.

It is noteworthy that, in our study, the saliva of *S. araneus* has been analysed for the first time. We did not record any significant effects of the salivary extract from the common shrew on the heart, nerve or muscle activity. Similarly, none of the fractionated compounds from *S. araneus* saliva displayed cardiotropic effects on the *T. molitor* heart. These results prove that *S. araneus* is not a venomous mammal. It seems there are no venomous species among the genus of *Sorex*. Suspicion remains concerning the cinereus shrew (*S. cinereus*), but its saliva has not been characterized thus far [9]. Lopez-Jurado and Mateo [26] suggested that the Canarian shrew (*Crocidura canariensis*) produces toxic saliva. However, as in the case of *S. cinereus*, the saliva of *C. canariensis* has not been analysed. More studies are required to prove the toxicity of saliva from these two species. It seems that smaller shrews, such as the common shrew, do not need to produce molecules with toxic activity in their saliva because they are unlikely to subdue large vertebrate prey, such as murid rodents or frogs. Our behavioural data provide experimental confirmation of this prediction as well (Kowalski et al., unpublished observations).

## Conclusions

Our results show that the venom of the water shrew displays stronger paralytic effects and lower cardioinhibitory activity. Therefore, we conclude that *N. fodiens* venom might be a useful tool in hunting and immobilizing larger prey. Additionally, for the first time, we separated and examined compounds from the water shrew venom and *S. araneus* saliva. Certain components from *N. fodiens* venom showed a weak positive impact on *T. molitor* heart contractile activity. However, we found that component(s) of sample no. 5 showed a strong negative chronotropic effect on the insect heart coupled with a reversible cardiac arrest. It enabled us to conclude that the water shrew can employ its venom to seize certain invertebrates, such as beetles or diplopods. Because none of the components from *S. araneus* saliva displayed toxic effects, we confirmed that the common shrew is not a venomous mammal. We suggest carrying out further analyses with a larger quantity of tissue material and more precise sample purification to identify proteins from the venom of *N. fodiens*. We are convinced that the present findings and further studies will enable us to better understand the role of venom in the animal world and mammal evolution in relation to toxicity. Moreover, because animal toxins can have applications in medicine and pharmacy [5, 43, 44], it is highly probable that our results also provide the opportunity for the design and development of new drugs, such as cardiac or neuromuscular blocking agents.

## Methods

### Animals

#### Shrews

Trapping sessions were performed in the suburbs of Poznań (western Poland) from April to October 2014–2016, excluding the coldest days. In total, we captured 25 water shrews and 25 common shrews. The captured shrews were transported to the laboratory. Next, the animals were placed separately into large (39 × 21 × 28 cm; 23 L) terraria equipped with bedding (a mixture of peat, moss and sand). Each terrarium contained a shelter (flowerpot) and a bowl with water. Food (mealworms, minced beef, earthworms and snails) and water were provided *ad libitum*. The shrews were kept in the breeding room under standard laboratory conditions (temperature: 21 °C; humidity: 65–70%; artificial photoperiod: 12 L:12D). After about a week, they were killed using approved methods to obtain their submandibular salivary glands.

#### Frogs

Frogs [15 common frogs (*Rana temporaria*) and 37 frogs from the genus of *Pelophylax* sp.] were captured by a net near ponds and small water tanks located in the Morasko district of Poznań (western Poland).

#### Insects

*Tenebrio molitor* L. adults (4 weeks old) were obtained from a culture maintained at the Department of Animal Physiology and Development, Adam Mickiewicz University, Poznań, Poland, as described previously [45].

### Venom/saliva collection and sample preparation

Shrews were killed by cervical dislocation, and their submandibular salivary glands were carefully isolated to obtain venom or saliva. Glands (*n* = 15; from each shrew species) designed for heart bioassays were transferred into 600 μl of insect Ringer’s solution (RS: 274 mM NaCl; 19 mM KCl; 9 mM CaCl; 5 mM glucose, and 5 mM HEPES, pH 7.0; bioassays with extract on the semi-isolated beetle heart) or 600 μl of frog Ringer’s solution (bioassays on frog organs). Glands (*n* = 10, from each shrew species) designed for chromatographic separation were transferred into 600 μl of methanol. Tissues were next homogenized, and samples were centrifuged at 10,000 × g and 4 °C for 30 min. The supernatants were collected, and the protein content was determined using a Direct Detect spectrometer (MERCK Millipore, Warsaw, Poland). Extracts with a final protein concentration of 1 μg/μl were used for bioassays.

### Chromatographic separation

Supernatants suspended in methanol were used for peptide analysis by reverse phase high-performance liquid chromatography (RP-HPLC). Separation was performed using a Dionex Ultimate 3000 chromatographic system comprising a dual pump programmable solvent module. Supernatants were analysed using a BioBasic-18 analytical column (5 μm, 150 × 4.6 mm; Thermo Scientific). The samples were eluted with a gradient of 5–60% acetonitrile (ACN)/0.1% TFA with a flow rate of 0.5 ml/min for 55 min. The eluent was monitored at 214 nm, and fractions were collected into 1.5-ml tubes. Next, ACN was evaporated, and samples were suspended in 70 μl of RS to determine the toxic activity of the separated components by performing bioassays on the semi-isolated *T. molitor* hearts.

### In vitro insect heart bioassay

Cardioactivity of the water shrew venom was assayed on the hearts of adult *Tenebrio molitor* and frogs. The microdensitometric method was used to measure the chronotropic (change in frequency) effect of samples on the semi-isolated heart of the adult beetle [46]. Briefly, *T. molitor* adults were decapitated, and the abdomen was removed. The ventral body wall of the abdomen was excised. The fat body, digestive system, and Malpighian tubules were removed from the abdomen. The final preparation consisted of the dorsal vessel (the heart), wing muscles, body wall muscles, and the dorsal cuticle. The heart preparations with a regular heartbeat were selected and superfused in RS. The superfusion chamber with the heart preparation was installed into the microdensitometer MD-100 (Carl Zeiss, Jena, Germany). An open perfusion system with an injection port 70 mm above the superfusion chamber was used. The flow rate of the fresh RS was 140 μl/min, and excess solution was removed from the superfusion chamber using chromatographic paper (Whatman No. 3, UK). All tested samples were applied at the injection port with a Hamilton syringe (10 μl). Many applications of samples could be sequentially assayed in a single preparation. The open system was designed to enable the addition of the samples avoiding changes in pressure. After 10 min of initial stabilization, the activity of the isolated heart was recorded for 30 s. Next, the sample was applied, and the heart activity was recorded for a further 1.5 min. This procedure was repeated with 5-min intervals for each tested sample.

Computer software (Larwa) developed at the Department of Animal Physiology and Development was used to record and analyse the cardiomyograms [47]. The activities of the analysed samples were presented as the percentage change in the frequency of the beetle heart contractions after sample application.

### In vitro frog heart bioassay

Frogs were decapitated, and the skin from the thorax was removed. The thorax wall was excised, and the pericardium was carefully removed (to not damage the heart). The mechanical responses of the spontaneously contracting, semi-isolated frog heart were measured by attaching one end of a thread to the apex of the heart using a clip and the other end to a force transducer (MLT 0420; ADInstruments, Australia). The contractile activity of the frog heart (the number of heart contractions/min) was recorded using a Power Lab 26T System (ADInstruments, Australia) attached to a computer equipped with Chart 5.5.4 software [48]. A given heart bioassay consisted of 5 steps:

1. measurement of the heart contractile activity (HCA) for 1 min immediately after the isolation of the frog heart;
2. measurement of HCA for 1 min after 40 μl of RS application on the semi-isolated frog heart (control test);
3. measurement of HCA for 1 min after 40 μl of venom application on the semi-isolated frog heart;
4. measurement of HCA for 1 min after step 3 (sometimes the effect of the venom appears in the 2^nd^ minute after venom application);
5. measurement of HCA for 1 min after 40 μl of RS application on the semi-isolated frog heart (to check the heart ability to recover).

This procedure was repeated with 5-min intervals for each tested sample. A maximum of 3 samples were applied on a single frog heart. The activities of the samples were presented as the percentage change in the frequency of the frog heartbeat after sample application.

### In vitro frog muscle bioassay

After frog decapitation, the skin from the hind part of the body and hind legs was removed. Next, the calf muscle was carefully isolated and transferred into a tub connected to the electrical stimulator incorporated into the Power Lab 26T unit. To stimulate the contraction of frog muscle, a 500-mV voltage was applied. The mechanical responses of the isolated muscle were measured by attaching one end of a thread to the tendon of muscle and the other end to a force transducer (MLT 0420; ADInstruments, Australia). The force of the muscle contraction was recorded using a Power Lab 26T System (ADInstruments, Australia) attached to a computer equipped with Chart 5.5.4 software [49]. A given muscle bioassay consisted of 5 steps (occurring with 1-min intervals):

1. measurement of the muscle contraction force (MCF) immediately after the isolation of the frog muscle;
2. measurement of MCF immediately after 40 μl of RS application on the isolated frog muscle (control test);
3. measurement of MCF immediately after 40 μl of venom application on the isolated frog muscle;
4. measurement of MCF 1 min after venom application;
5. measurement of MCF after 40 μl of RS application (to check the muscle ability to recover).

This procedure was repeated with 5-min intervals for each tested sample. A maximum of 3 samples were applied on a single frog calf muscle. The activities of samples were presented as the percentage change in the force of the muscle contraction after sample application.

### In vitro frog nerve bioassay

After frog decapitation, the skin from the hind part of the body and hind legs was removed. The ventral wall of the body was excised, and the viscera were removed. Next, the nerve trunk was looped, and the sciatic nerve was carefully isolated (to not damage it) and transferred into a tub connected to the electrical Power Lab 26T as described previously. To stimulate the conduction velocity of the frog nerve, a 500-mV voltage was applied. The conduction velocity was recorded using the Power Lab 26T System (ADInstruments, Australia) attached to a computer [50]. The protocol of a given nerve bioassay was the same as that of a muscle assay.

This procedure was repeated with 5-min intervals for each tested sample. A maximum of 3 samples were applied on a single frog nerve. The activities of the samples were presented as the percentage change in the conduction velocity of the frog nerve after sample application.

### Protein identification

Proteomic analysis was carried out at the Mass Spectrometry Laboratory, Institute of Biochemistry and Biophysics, Polish Academy of Sciences, Warsaw. Peptides from the extract of *N. fodiens* venom and *S. araneus* saliva were analysed by liquid chromatography coupled to tandem mass spectrometry LC-(MS-MS/MS) using a Nano-Acquity LC system (Waters, Milford, Massachusetts, USA) and an OrbitrapVelos mass spectrometer (Thermo Electron Corp., San Jose, CA). Before performing the analysis, the proteins were subjected to an ion-solution digestion procedure. Proteins were (1) reduced with 50 mM TCEP for 30 min at 60 °C, (2) alkylated with 200 mM MMTA for 30 min at room temperature and (3) digested overnight with trypsin (sequencing Grade Modified Trypsin - Promega V5111). Next, the samples were applied to an RP-18 precolumn (nanoACQUITY Symmetry® C18 - Waters 186,003,514) using water containing 0.1% TFA as a mobile phase and were transferred to a nano-HPLC RP-18 column (nanoACQUITY BEH C18 - Waters 186,003,545). The samples were eluted with a gradient of 0–35% acetonitrile in the presence of 0.05% formic acid with a flow rate of 250 nl/min for 180 min. The column was directly coupled to the ion source of the spectrometer working within data dependent on the MS to MS/MS switch. To ensure a lack of cross contamination from previous samples, each analysis was preceded by a blank run. The proteins were identified by a Mascot Search (Matrix Science, London, UK) against the SwissProt and NCBInr databases. The search parameters were as follows: type of search: MS/MS Ion Search; enzyme specificity: trypsin; fixed methylthio modification of cysteine; variable modifications: methionine oxidation; mass values: monoisotopic; protein mass: unrestricted; peptide mass tolerance: 30 ppm; fragment mass tolerance: 0.1 D; number of missed cleavage sites allowed: 1; instrument type: HCD. Peptides with Mascot scores exceeding the threshold value of *p* < 0.05 were considered positively identified.

### Data analysis

All data were presented as the mean values ± SEM (standard error of the mean) of the indicated number of replicates (n). The mean differences between the treatment and control group were determined using paired Student’s *t*-test or Wilcoxon signed-rank test. To indicate statistically significant differences between *N. fodiens* venom and *S. araneus* saliva activity, unpaired Student’s *t*-test or Mann-Whitney *U*-test was performed. Non-parametric tests (Wilcoxon signed-rank and Mann-Whitney) were performed when the datasets of non-normal distributions were compared. To counteract the problem of multiple comparisons, Bonferroni correction was performed. All statistical analyses were carried out using R software [51]. Differences were considered as statistically significant for *p-*values less than 0.05.

## Additional file

Additional file 1: Table S1. Cardiotropic effects of compounds from *Neomys fodiens* venom and *Sorex araneus* saliva on the *Tenebrio molitor* heartbeat frequency (* results are presented as percentage change in the heartbeat frequency ± SEM; n - number of replicates, *W* - Wilcoxon test value, *p’* - *p*-value after Bonferroni correction). (DOCX 19 kb)
