# Supplementary material for: Evaluation of the physiological activity of venom from the Eurasian water shrew Neomys fodiens
Source: Front Zool. 2017 Sep 30;14:46. doi: 10.1186/s12983-017-0230-0 (PMC5622582; doi:10.1186/s12983-017-0230-0)
Supplement: Additional file 1: Table S1. — Cardiotropic effects of compounds from Neomys fodiens venom and Sorex araneus saliva on the Tenebrio molitor heartbeat frequency (* results are presented as percentage change in the heartbeat frequency ± SEM; n - number of replicates, W - Wilcoxon test value, p’ - p-value after Bonferroni correction). (DOCX 19 kb) [file 12983_2017_230_MOESM1_ESM.docx]

**Table S1.** Cardiotropic effects of compounds from *Neomys fodiens* venom and *Sorex araneus* saliva on the *Tenebrio molitor* heartbeat frequency (* results are presented as percentage change in the heartbeat frequency ± SEM; n - number of replicates, *W* - Wilcoxon test value, *p’* - *p*-value after Bonferroni correction)

| Fraction no. | | | % ± SEM* | n | *W* | *p* | *p’* |  |
| --- | --- | --- | --- | --- | --- | --- | --- | --- |
|  | | ***Neomys fodiens*** | | | | | | |
| 5 | -43.70 ± 9.40 | | | 6 | 60.0 | 0.0002 | 0.005 |  |
| 6 | -0.37 ± 1.42 | | | 7 | 25.5 | 0.21 | 1.00 |  |
| 8 | -1.44 ± 0.91 | | | 6 | 22.0 | 0.42 | 1.00 |  |
| 10 | -0.27 ± 0.57 | | | 6 | 17.5 | 0.19 | 1.00 |  |
| 12 | -0.36 ± 0.69 | | | 7 | 21.0 | 0.19 | 1.00 |  |
| 15 | 0.89 ± 0.32 | | | 7 | 6.00 | 0.003 | 0.075 |  |
| 16 | -0.60 ± 1.02 | | | 7 | 25.0 | 0.36 | 1.00 |  |
| 17 | 0.76 ± 0.24 | | | 7 | 6.00 | 0.005 | 0.125 |  |
| 19 | 0.90 ± 0.50 | | | 7 | 6.00 | 0.003 | 0.075 |  |
| 21 | 0.65 ± 0.79 | | | 7 | 13.0 | 0.03 | 0.75 |  |
| 22 | 1.88 ± 1.09 | | | 6 | 3.50 | 0.005 | 0.125 |  |
| 23 | -0.01 ± 0.53 | | | 6 | 11.0 | 0.06 | 1.00 |  |
| 24 | 0.06 ± 1.01 | | | 7 | 24.0 | 0.30 | 1.00 |  |
| 25 | 0.61 ± 0.67 | | | 7 | 15.0 | 0.06 | 1.00 |  |
| 27 | 1.12 ± 0.88 | | | 6 | 6.00 | 0.007 | 0.175 |  |
| 28 | 0.62 ± 0.45 | | | 7 | 5.00 | 0.004 | 0.1 |  |
| 29 | 0.58 ± 0.90 | | | 7 | 12.0 | 0.03 | 0.75 |  |
| 31 | 5.55 ± 1.55 | | | 6 | 1.00 | 0.0005 | 0.012 |  |
| 32 | 1.04 ± 0.91 | | | 7 | 13.0 | 0.003 | 0.075 |  |
| 34 | 5.62 ± 0.74 | | | 7 | 0.00 | 0.0001 | 0.002 |  |
| 38 | 4.09 ± 0.81 | | | 6 | 1.00 | 0.0005 | 0.012 |  |
| 39 | 2.82 ± 1.66 | | | 7 | 7.00 | 0.005 | 0.125 |  |
| 40 | 4.56 ± 1.90 | | | 6 | 2.00 | 0.0009 | 0.022 |  |
| 42 | 1.10 ± 1.31 | | | 7 | 19.0 | 0.13 | 1.00 |  |
| 43 | 2.69 ± 1.63 | | | 6 | 4.00 | 0.005 | 0.125 |  |
|  | | ***Sorex araneus*** | | | | | | |
| 17 | -0.33 ±0.55 | | | 6 | 12.5 | 0.41 | 1.00 |  |
| 23 | 1.58 ± 2.01 | | | 6 | 13.0 | 0.47 | 1.00 |  |
| 26 | -1.78 ± 0.64 | | | 7 | 24.0 | 0.71 | 1.00 |  |
| 27 | -0.78 ± 0.77 | | | 7 | 16.5 | 0.56 | 1.00 |  |
| 28 | -2.33 ± 1.02 | | | 6 | 21.0 | 0.68 | 1.00 |  |
| 29 | 2.58 ± 1.14 | | | 6 | 6.00 | 0.05 | 0.8 |  |
| 30 | 0.64 ± 0.61 | | | 7 | 9.50 | 0.11 | 1.00 |  |
| 31 | 0.83 ± 0.71 | | | 6 | 10.0 | 0.22 | 1.00 |  |
| 32 | -0.58 ± 0.84 | | | 6 | 15.0 | 0.68 | 1.00 |  |
| 43 | -0.42 ± 0.44 | | | 7 | 15.0 | 0.42 | 1.00 |  |
| 44 | -0.71 ± 0.76 | | | 7 | 17.0 | 0.60 | 1.00 |  |
| 45 | -0.08 ± 0.37 | | | 6 | 12.0 | 0.37 | 1.00 |  |
| 46 | -0.42 ± 0.71 | | | 7 | 15.0 | 0.42 | 1.00 |  |
| 47 | 0.66 ± 0.64 | | | 6 | 10.0 | 0.21 | 1.00 |  |
| 48 | 0.57 ± 0.27 | | | 7 | 10.0 | 0.12 | 1.00 |  |
| 49 | 0.42 ± 0.48 | | | 6 | 9.00 | 0.17 | 1.00 |  |
